# Supplementary material for: Super-resolution imaging of light–matter interactions near single semiconductor nanowires
Source: Nat Commun. 2016 Dec 20;7:13950. doi: 10.1038/ncomms13950 (PMC5187462; doi:10.1038/ncomms13950)
Supplement: Supplementary Information — Supplementary Figures, Supplementary Notes and Supplementary References. [file ncomms13950-s1.pdf]

SUPPLEMENTARY FIGURES

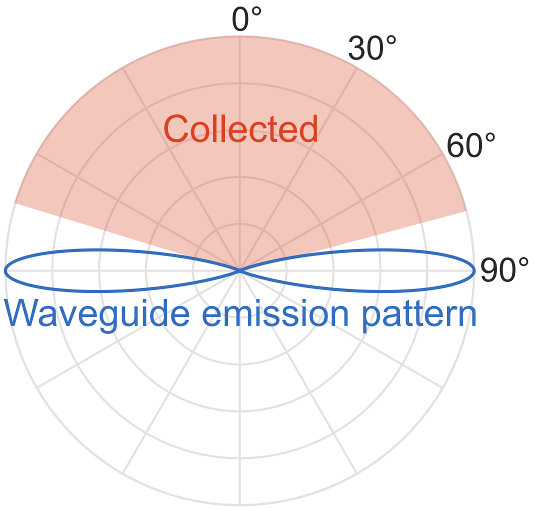

Supplementary Figure 1. **Silicon nanowire waveguide emission.** Normalized polar emission distribution for the HE_{11} waveguide mode of a 75 nm diameter silicon nanowire oriented along the 90° orientation, and the collection angle of a 0.965 numerical aperature objective.

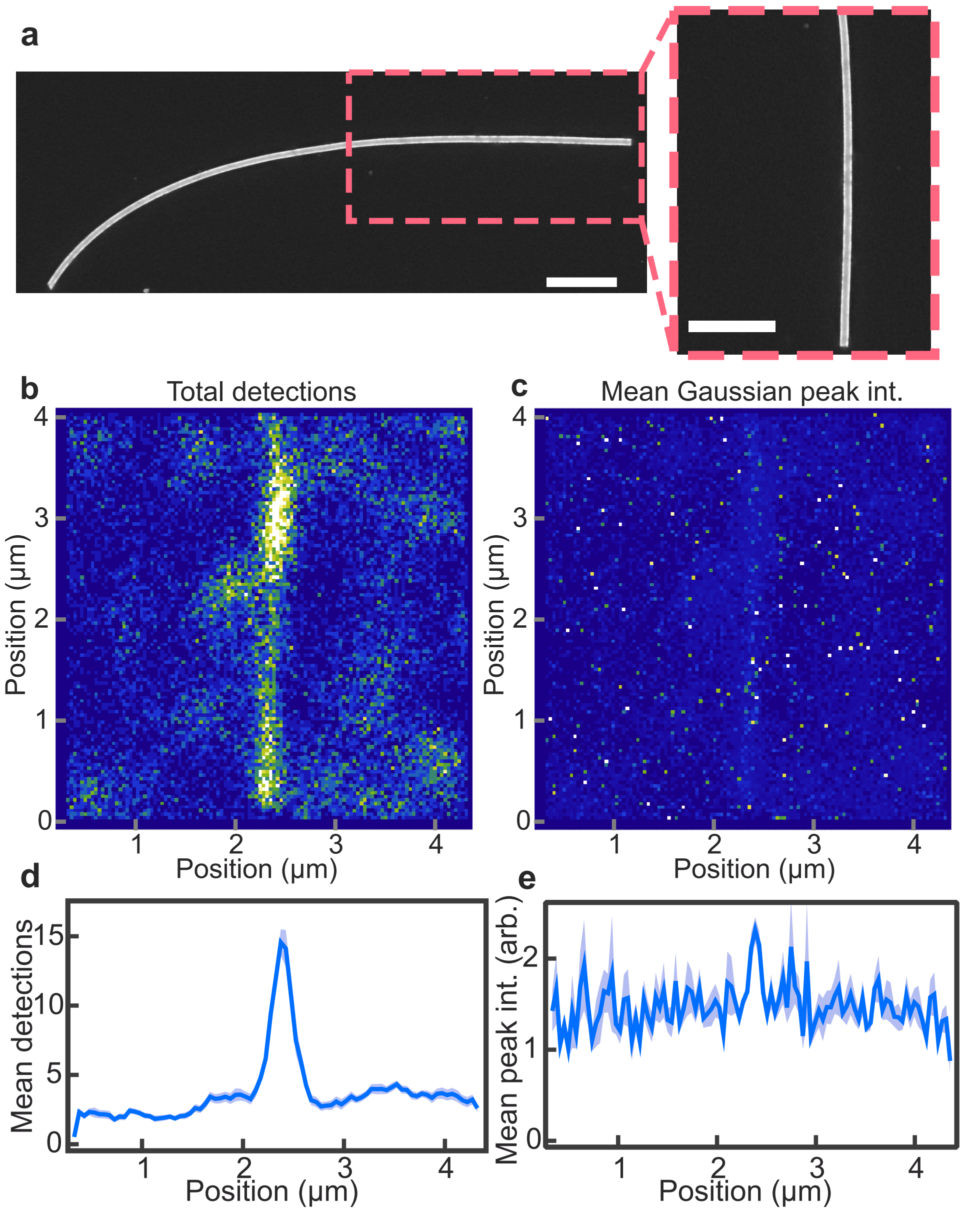

Supplementary Figure 2. **Control experiments on index-matched nanostructures.** a, SEM image of measured silicon oxide nanowire with the studied (non-curved) region highlighted (scale bars = 1 μm). b, c, 2-dimensional super-resolution maps of the total detected fluorescence events in a given pixel during the 30 minute measurement, and their mean Gaussian peak intensity (respectively) for the region denoted in a. Pixel size is 40 nm × 40 nm. d, e, Line scans of the full region in the above images integrated along the wire, of the mean number of detection events and Gaussian peak intensity, respectively, showing increased localizations on the wire surface but no change in fluorescence intensity. The thin grey region corresponds to standard error of line scan integration.

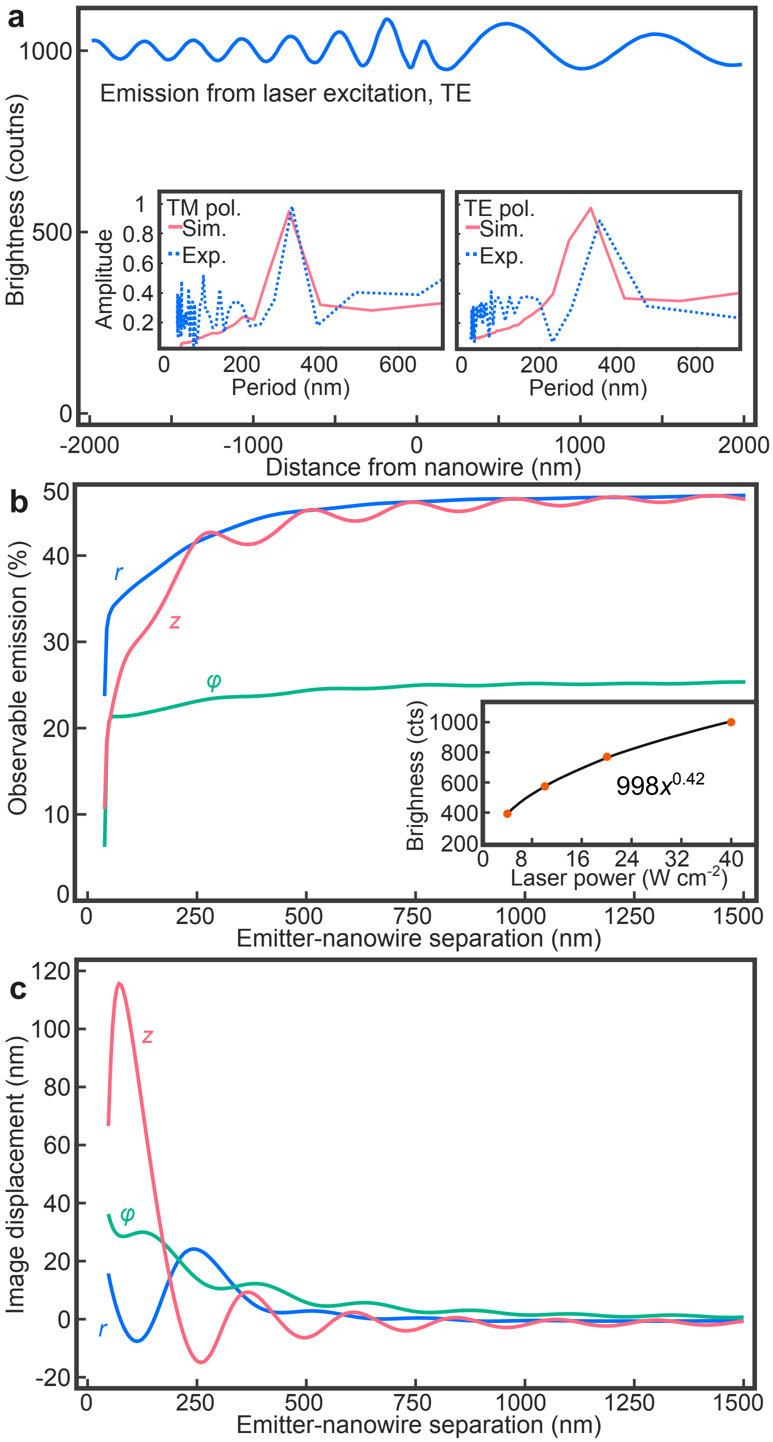

Supplementary Figure 3. **Components of simulated dipole interactions.** a, Laser excitation contribution to dye brightness, showing the non-uniform field produced by the laser interference with scattering the nanowire. The asymmetry is due to the laser entering the system at an oblique angle. Inset, Fourier analysis comparing the intensity of oscillation periods of experimental and simulated left hand side regions, for TM (electric field oriented parallel to the nanowire axis) and TE (electric field perpendicular to the nanowire axis) collection polarizations. b, Observable point spread function (PSF) peak brightness due to scattering and PSF distortion, for three dipole orientations r, ϕ and z, measured as the collection in a 0.967 numerical aperture objective, normalized to the total dipole emission. Inset, Fit between the set laser power and empirically measured dye brightness in our experiments, used to map the simulated excitation power to a simulated brightness. Brightness is measured in the total number of photon counts obtained from a fluorescence event. c, Displacement of the PSF image from the true dipole emitter location, due to distortions of the PSF. All distances are taken to the nanowire centre.

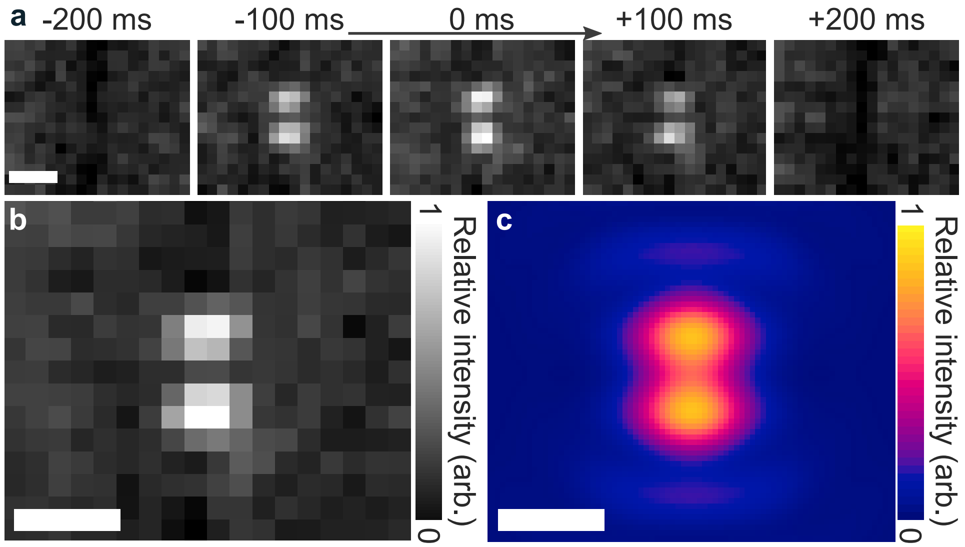

Supplementary Figure 4. **Distorted point spread function comparison.** a, Time series of experimental point spread function at nanowire location, showing two-lobed PSF activating and bleaching. b, Time average of central three images in a, compared to c, a simulated point spread function of a z-aligned dipole 4 nm from the surface of the simulated wire. All scale bars are 500 nm.

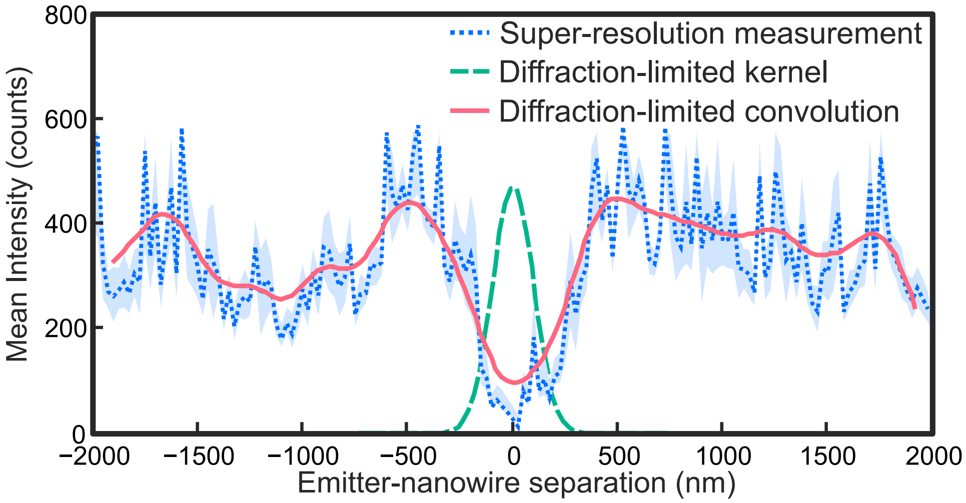

Supplementary Figure 5. **Loss in information from diffraction-limited resolution.** Comparison of measured super-resolution experimental data (blue), to that convoluted with a diffraction-limited width Gaussian kernel, $\sigma \approx 135$ nm (green dashed line), approximating the highest resolution obtainable measurement without super-resolution techniques (solid pink line). Distances are take to the centre of the nanowire. The thin grey region corresponds to standard error of line scan integration.

SUPPLEMENTARY NOTES

Supplementary Note 1 – Waveguide emission, absorption and scattering

For the 75 nm diameter silicon nanowire investigated in this study, the decay length of the waveguide mode for this geometry is close to 800 µm, and so absorption can be ignored. Additionally, the scattering cross section for a TM polarized plane wave under normal incidence is ∼70 times the absorption cross section (255 versus 3.70 nm)[1], indicating that indeed these resonances do not lead to significant absorption but do lead to very strong scattering.

In Supplementary Fig. 1 we show the finite-difference time-domain (FDTD) calculated emission pattern from the nanowire HE_{11} waveguide mode. The power emitted from the waveguide at the end facet of the nanowire is emitted directively in the orientation of the nanowire axis, due to the very weak confinement of the mode. For the collection angle of our objective (shaded red) we see that almost none of this power would be collected, causing emission from waveguide modes to be undetected (indistinguishable from absorption) in our measurements.

Supplementary Note 2 – Additional experimental analysis details

For these measurements, the settings in the ThunderSTORM software[2] found to give the best balance between avoiding false-positive signals while maintaining localization of dim events consisted of utilizing a wavelet transform with a B-spline order of 3 and scale of 2.0 for initial image filtering, local maximum peak intensity thresholding using an F1 score scaling of 0.8, and a weighted least-squares regression to an elliptical Gaussian profile with a fitting radius of 3 pixels assuming an initial guess of $\sigma = 1.4$ pixels for sub-pixel localization. Drift corrections are calculated by the localization of gold nanoparticles, and fluorophore molecules within a window of 80 nm that survive for multiple frames are merged. For additional information see Supplementary Reference 2.

Distortions of the point spread function (PSF) by the nanowire, combined with a temporally fluctuating, spatially non-uniform background level (arising from out of plane fluorescence events) would cause significant artifacts in measurements of the total photon count from a fluorophore, especially for events close to the nanostructure (high distortion)[3], and dim events on bright backgrounds (falsely fit as extremely extended Gaussian distributions). To avoid this, the peak of the Gaussian fit to the PSF is used in lieu of the integrated photon count in both the experimental and computational analysis. While this allows direct mapping between experiment and theory, it does exaggerate the influence of scattering relative to a (background-free) measurement where all photons within the NA of the system are accounted for. For the purpose of analysis and comparison to theoretical calculations, the Gaussian height is multiplied by the square of the measurement's mean Gaussian width to return to a unit of counts (for our 95 ms integration time), corresponding to a distortion-free PSF.

Detected localizations are then averaged over a pixel size of 25 nm × 25 nm, in 2- and 1-dimensional traces, as shown in Fig. 2e-h of the main text. Individual localizations observed for more than 15 frames, with a brightness of more than four standard deviations above the mean, or with an uncertainty beyond four pixels (100 nm, or twice the mean) are discarded to reduce the presence of clustered fluorophores which can not be localized individually.

The 25 nm × 25 nm bin size was chosen as an optimal trade-off between increasing the size to limit the occurrence of zero pixels (bins without data points) in the background (zero pixels in the trough region are likely meaningful, while those in the background are not), and decreasing the pixel size to improve the resolution of the measurements and visibility of features of the trough and oscillations, for example. Additional analysis was done to confirm that the converged (no zero pixels in the background) brightness of the background of both TM and TE polarization measurements match the simulated values (∼875 and ∼500 counts, respectively).

Supplementary Note 3 – Oxidized nanowire control measurements

As a control measurement for our experiments on semiconductor nanowire systems, it is necessary to ensure that the observed phenomena in the silicon wire measurements are indeed purely optical. Insomuch, we perform super-resolution point accumulation for imaging in nanoscale topography (PAINT)-intensity measurements on fully oxidized silicon wires in order to preclude the presence of physical influences (for example, blocking of the dye by the wire). The near-zero absorption and index-matching between the oxidized wires (n≈1.5), glass substrate (n≈1.5) and solution (n≈1.4), provide an ideal control, as there should be virtually no change in the measured intensity near and away from the wires.

Wires are oxidized from a subset of the same growth substrate as used in the measurements in the main text, using wet oxidation of the nanowires for 7 hours at 1000°C. Full oxidation is confirmed using scanning transmission electron microscopy (STEM) as well as energy-dispersive X-ray spectroscopy (EDX).

The results of the super-resolution imaging of the 100 nm diameter silicon oxide wire are shown in Supplementary Fig. 2. A SEM image of the measured nanowire is shown in a, with the dashed red demarcated region indicating the area probed in the PAINT-intensity measurements. In b and c, we show 2D plots of the wire, here with the former showing the number of fluorescence events localized within a 40 nm × 40 nm box size, while c shows the peak intensity (unpolarized); d ande show line traces resulting from averaging along the integration box shown in b and c, respectively. The results here are as expected: while we observe a larger number of localizations on the wire, the average intensity of the localization peaks remains unperturbed by the presence of the wire. This increased localization count near the wire is likely due to the increased surface area around the wire region, although the width is somewhat exaggerated by a slight curvature in the wire, as well as possible clustering adjacent to the wire.

These measurements provide clear evidence that the phenomena in the silicon wire measurement are indeed purely optical, and additionally suggests that fluorophore localizations are occurring on stochastically adsorbed fluorophores.

Supplementary Note 4 – Additional calculation and simulation details

The calculations for a given simulated fluorophore begin by computing the influences of the diffraction of the excitation laser with the nanowire, as plotted in Supplementary Fig. 3a. The asymmetry of the response is due to the laser entering the system at a 30° angle to the surface (to minimize reflection back into the collection system in the experimental setup). The inset plots compare the observed experimental oscillations in the left hand side of Fig. 2g,h of the main text to the simulated response, showing good agreement between the dominant period visible in the frequency-domain analysis.

The local electric field, E, is computed with Lorenz-Mie theory[1] calculations, and the scaling is determined by an empirical fit of laser power to dye brightness done in the absence of any structure (inset of Supplementary Fig. 3b), also accounting for the angle between the excitation source and dipole. This provides us with the equivalent free-space brightness of a fluorophore as a function of position to the nanowire. This also permits a measurement of the degree of saturation of our fluorophore pump intensity, indicating an intermediate regime of saturation.

Near-field and far-field (asymptotic) Green's function calculations are performed for a dipole interacting with an infinitely long nanowire (a reasonable approximation due to the large aspect ratio of the measured wires), at distances from 5 to 1500 nm from the nanowire surface, at a resolution of 3 nm. Additionally, within 500 nm of the surface of the wire, a range of 20 angles from $[-\frac{\pi}{2}, \frac{\pi}{2}]$ (due to symmetry) with respect to the observation plane are used for the asymptotic calculations.

The LDOS of the system is computed from the near-field Green's function calculations, taking the imaginary part of the inhomogeneous Green's function at the location of the dipole, providing the radiative decay rate[4], $\mathit{\Gamma}$. The integration of the Green's function is performed using an adaptive Gauss-Kronrod quadrature[5] along an integration path deformed to avoid branch cuts present on the real axis, implemented in Julia[6]. Knowing the free-space quantum efficiency, η_0, of 0.57 (Abberior), the modification of the environmentally-modified quantum efficency, η, from the LDOS can be calculated[7] as $\eta = \mathit{\Gamma}/(\mathit{\Gamma} + 0.72)$. At full saturation, the emission would also be proportional to the rate enhancement, $\mathit{\Gamma}$, whereas in the linear response regime (far below saturation) emission would be independent of rate. We estimate the influence of the intermediate saturation of our measurements by incorporating the slope of the saturation response, s' (0.42–0.63, depending on dipole polarization with respect to the source) as an exponent in $\mathit{\Gamma}$.

As shown in Supplementary Fig. 3b, we compute the scattering of the dipole emission with the nanowire, and the projection of this emission to the image plane. Asymptotic Green's function calculations[4] involve computing the electric field at large distances from the origin over a quarter spherical surface (again due to symmetry), yielding an equivalent momentum-space field distribution. This distribution is truncated using a cut-off determined from the NA of 0.967 of our experimental system.

We then use a discrete Fourier transform of the complex momentum-space electric field, E, to compute the real-space intensity ($|E|^2$) distribution at the image plane (our photodetector)[7]. From this image, we fit a Gaussian function to the global maximum of the PSF, providing the equivalent detectable peak brightness to that of a given dipole (fluorophore) emission event in our experimental measurements. As the asymptotic Green's function already inherently includes the rate modification of the source by the local environment, in order to avoid double-counting we must transform the obtained aplitude, A, into a rate-normalized amplitude, A^*, through $A^* = A/\mathit{\Gamma}$, as the rate modification ($\mathit{\Gamma}$) is taken into account (with the known free-space quantum efficiency and saturation levels) elsewhere.

The combination of these influences allows for the calculation of emission brightness,

$$B(r, \phi, \theta) = a(|E(r, \phi, \theta)|^2)^b \cdot \mathit{\Gamma}^{(1-s')}(r, \theta) \cdot \eta(r, \theta) \cdot A^* \cdot P(\theta) \tag{1}$$

where r is the radial distance to the nanowire surface, ϕ is the angle relative to the substrate surface, θ is the dipole polarization angle, and P is the weighting of the linear measurement polarization (TM/TE; effectively selecting for

dipole orientations). The prefactor a and exponent b are empirical fits from the fluorophore brightness response measurements (Supplementary Fig. 3b, inset), yielding a B in units of counts, and directly comparable with experiment.

Finally, in Supplementary Fig. 3c, we plot the peak displacement of the dipole image from its free-space position. This is simply calculated from the location of the peak of the Gaussian fit described in the preceding paragraph, with the significant displacements occurring due to the distortion of PSFs resulting from interference of the dipole emission with the light scattered by the nanowire.

An example of such a distortion is shown in Supplementary Fig. 4. Although the distortion shown here does not contribute to a displacement in the radial direction, the distinct two-lobe structure makes it most obvious to observe and thus confirm the agreement between experiments and simulations.

The simulations additionally incorporate the known distributions of dye location uncertainty, u. This allows us to generate simulated traces of expected fluorophore luminescence collection with respect to our simulated wire location, equivalent to our experimental measurements, giving a final position-dependent brightness as

$$B^*(x = r * \sin\phi + u + d) = B(r, \phi, \theta) \tag{2}$$

where d is the displacement from the distortion of the PSF, u is stochastically distributed with the measured uncertainty of the dye, and x is the projection of the dipole position onto the lateral position relative to the nanowire.

As in experiments, the produced data for all virtual fluorescence events is binned into pixels of 25 nm × 25 nm. A single fitting parameter of the noise floor, responsible for limiting the dimmest events which can be localized, is incorporated for the TE measurements, fixed, and used similarly for the TM measurements, producing the final traces shown in Fig. 2g,h in the main text.

Supplementary Note 5 – Need for super-resolution

In Supplementary Fig. 5 we demonstrate the necessity of sub-diffraction-limited localization of emission events to these measurements. We see that in the TE polarized line trace, all fine features within the dim region surrounding the wire are lost when the experimental data is convoluted with a diffraction-limited Gaussian kernel (standard deviation of $\sigma = 135$ nm). Furthermore, the 320 nm period peak in the left side oscillations (due to the laser-nanowire interference) of both the TM and TE polarization traces is significantly attenuated, demonstrating further loss of information. Together, these indicate that under diffraction-limited measurements the confirmation of our modelling to our experimental results would be unfeasible, and thereby solidify the need for super-resolution localization.

Supplementary Note 6 – Diffusion calculations

Lateral diffusion of the dye during measurement contributes to a bright background. This is particularly from out of focus fluorophore events, whereas the low uncertainty of the localized events (mean squared error = 50 nm^2) suggests that fluorophores are being localized only when adsorbed on the glass or nanowire surface. This is supported by Stokes-Einstein equation[8] estimates of the fluorophore mean squared displacement (MSD) during the acquisition time on the order of 10 µm^2, accounting for our calculated solution viscosity[9].

Additionally, we estimate that the rotational diffusion within the fluorescence lifetime (∼4 ns) to be low (MSD = 0.5 rad^2)[8], and thus approximate molecules as having a fixed, random orientation in our modelling. This is corroborated by the relative intensities of the TM and TE polarization measurements in Fig. 2g,h of the main text, as the 30° angle of the nanowire with respect to the excitation polarization contributes (along with the difference in localization events) to the observed difference in average intensity.

SUPPLEMENTARY REFERENCES

[1] Bohren, C. F. & Huffman, D. R. *Absorption and Scattering of Light by Small Particles* (Wiley-VCH Verlag GmbH, 1998).
[2] Ovesny, M., Krizek, P., Borkovec, J., Svindrych, Z. & Hagen, G. M. ThunderSTORM: A comprehensive ImageJ plug-in for PALM and STORM data analysis and super-resolution imaging. *Bioinformatics* **30**, 2389–2390 (2014).
[3] Su, L. *et al.* Visualization of molecular fluorescence point spread functions via remote-excitation stochastic switching fluorescence microscopy (RE-SSFM). *Nat. Commun.* **6**, 6287 (2015).
[4] Tai, C.-T. *Dyadic Green functions in electromagnetic theory* (1994).
[5] Laurie, D. P. Calculation of Gauss-Kronrod quadrature rules. *Math. Comput.* **66**, 1133–1146 (1997).
[6] Bezanson, J., Karpinski, S., Shah, V. B. & Edelman, A. Julia: A fast dynamic language for technical computing. *Preprint at http://arxiv.org/abs/1209.5145* (2012).
[7] Novotny, L. & Hecht, B. *Principles of Nano-Optics* (Cambridge University Press, 2006).
[8] Cooper, A. *Biophysical Chemistry* (The Royal Society of Chemistry, 2004).
[9] Wagner, H. L. The Mark-Houwink-Sakurada Relation for Poly(Methyl Methacrylate). *J. Phys. Chem. Ref. Data* **16**, 165–173 (1987).
